# Choosing the right molecular machine learning potential

**DOI:** 10.1039/d1sc03564a

**Published:** 2021-09-15

**Authors:** Max Pinheiro, Fuchun Ge, Nicolas Ferré, Pavlo O. Dral, Mario Barbatti

**Affiliations:** Aix Marseille University, CNRS, ICR Marseille France max.pinheiro-jr@univ-amu.fr mario.barbatti@univ-amu.fr; State Key Laboratory of Physical Chemistry of Solid Surfaces, Fujian Provincial Key Laboratory of Theoretical and Computational Chemistry, Department of Chemistry, College of Chemistry and Chemical Engineering, Xiamen University China dral@xmu.edu.cn; Institut Universitaire de France 75231 Paris France

## Abstract

Quantum-chemistry simulations based on potential energy surfaces of molecules provide invaluable insight into the physicochemical processes at the atomistic level and yield such important observables as reaction rates and spectra. Machine learning potentials promise to significantly reduce the computational cost and hence enable otherwise unfeasible simulations. However, the surging number of such potentials begs the question of which one to choose or whether we still need to develop yet another one. Here, we address this question by evaluating the performance of popular machine learning potentials in terms of accuracy and computational cost. In addition, we deliver structured information for non-specialists in machine learning to guide them through the maze of acronyms, recognize each potential's main features, and judge what they could expect from each one.

## Abstract 2

This article provides a lifeline for those lost in the sea of the molecular machine learning potentials by providing a balanced overview and evaluation of popular potentials.

## Graphical abstract

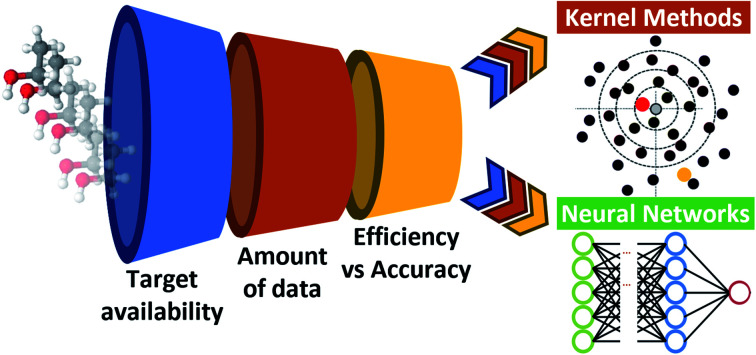

## Introduction

Potential energy surface (PES) is the central concept in chemistry and materials science for understanding the processes on an atomistic scale. This function of the atoms' spatial coordinates can be interpreted as a multidimensional energy landscape that drives the continuous kinetic transformations of atomistic systems and determines their propensity to undergo chemical reactions. Once we determine the PES, we can calculate relevant observables such as vibrational spectra and reaction rates.^1^ However, the considerably large number of computationally costly quantum-chemistry calculations required to construct reliable PESs and the lack of flexibility by empirical fitting methods pose an enormous challenge. Hence, the development of efficient methods to generate high-quality PES for molecules and materials has been a cornerstone of computational chemistry research.

Recently, machine learning (ML) has emerged as a promising approach that is rocking the foundations of how we simulate molecular PES.^2–9^ Built on statistical principles, ML-based PESs, or more simply ML potentials (MLPs), aim to identify an unbiased predicting function that optimally correlates a set of molecular structures with the given target energies and, often, forces used as training data. (The force acting on the nuclei is the negative of the PES gradient.) Owing to its generalization capabilities and fast prediction on unseen data, MLPs can be explored to accelerate minimum-energy^10–15^ and transition-state structure search,^13,16,17^ vibrational analysis,^18–21^ absorption^22,23^ and emission spectra simulation,^24^ reaction^13,25,26^ and structural transition exploration,^27^ and ground-^3–9^ and excited-state dynamics propagation.^28,29^

A blessing and a curse of ML is that it is possible to design, for all practical purposes, an infinite number of MLP models that can describe a molecular PES. These MLP models are usually built from two main components: the ML algorithm and its input X, the molecular descriptor.^3–9^ By choosing a specific ML algorithm, we can restrict the hypothesis space of mapping functions to a searchable size. These mapping functions used in the learning process can be conveniently expressed as either parametric or nonparametric ML algorithms.^30^ Parametric algorithms are built on the assumption that the mapping function has a predefined functional form with a fixed number of parameters independently of the number of training samples. Neural networks (NNs) is a typical example. In turn, nonparametric algorithms do not make such a strong assumption. Thus, their complexity and number of parameters increase for more training data. Such is the case of kernel methods^31^ (KM) like the kernel ridge regression (KRR) and Gaussian process regression (GPR). The ML algorithm's hyperparameters introduce another layer of flexibility. NN-based parametric methods can be tuned by optimizing the NN's architecture (defined by the number of nodes and hidden layers), the activation function, and learning-rate criteria. Nonparametric methods can be tuned by searching the optimal functional form (*e.g.*, KRR can use linear, polynomial, Gaussian, Matérn kernel functions, among others). In both cases, there are still other hyperparameters related to model regularization that can be tuned to improve further the model's performance. Still, the final accuracy of the MLP model crucially depends on the choice of the descriptor used as input for the ML algorithm.^3–8^ MLPs usually follow the path of classical molecular mechanics potentials by using structural descriptors derived from 3D geometries defined by atomic positions and nuclear charges. A wide variety of descriptors has been developed,^3–8^ differing mainly about the approach adopted to characterize the chemical environment.

All these technological advances and their successful applications indicate that ML techniques in quantum chemistry are now reaching a mature stage, as is evidenced by the recent appearance of many specialized “black-box” software.^32–39^ These programs have been designed not only to predict chemical properties of compounds but also to be easily interfaced with other software to perform follow-up tasks like spectrum or dynamics simulations. On the other hand, all this variety of available MLPs makes it a formidable task to choose an MLP model among the rapidly expanding sea of published models. It is also becoming increasingly difficult even for specialists to gauge their performance and follow all their technical details. Thus, various reviews, perspectives, tutorials, and books have been published at an ever-increasing pace to survey state-of-the-art MLPs (just a small selection of reviews are in ref. 3–9). Complementary to these studies, an effort was made to benchmark^40^ the performance (accuracy and efficiency) of MLPs with respect to energy predictions by focusing specifically on the algorithm component of the models. Nevertheless, none of the previous works has addressed the challenging problem of providing a roadmap to MLPs and comparing the performance of established methods, especially those implemented in stand-alone software packages,^32–39^ in terms of accuracy and computational resources.

In this article, we aim at providing a resource for more straightforward navigation in this sea of MLPs. Here, we pursue three goals. First, we want to shed light on the miscellany of methods allowing researchers to guide themselves amidst the multitude of acronyms. Second, we aim to provide a guideline to assess the performance expected from the most promising and popular MLP types. Third, we also aim to provide qualified information derived from representative tests to aid researchers in making an informed decision when choosing an MLP for a particular molecular system or application. Taken together, these goals will help to advance our understanding of the intricate relations between data distribution, model architecture, and performance. We also briefly discuss how the accuracy of MLP models may deteriorate when considering increasingly large and complex molecular systems.

We emphasize that we are not attempting to answer the question of what is the most accurate MLP among all available models. Such an attempt would be meaningless as it is like shooting at a moving target because of the growing diversity of MLPs, the variety of possible applications, and the rapidly expanding amount of data available for benchmarking. In addition, some published MLP models have no available program or have poorly documented programs, or, in the worst case, the published results are simply irreproducible due to continuous change in the code version.

**Fig. 1:**
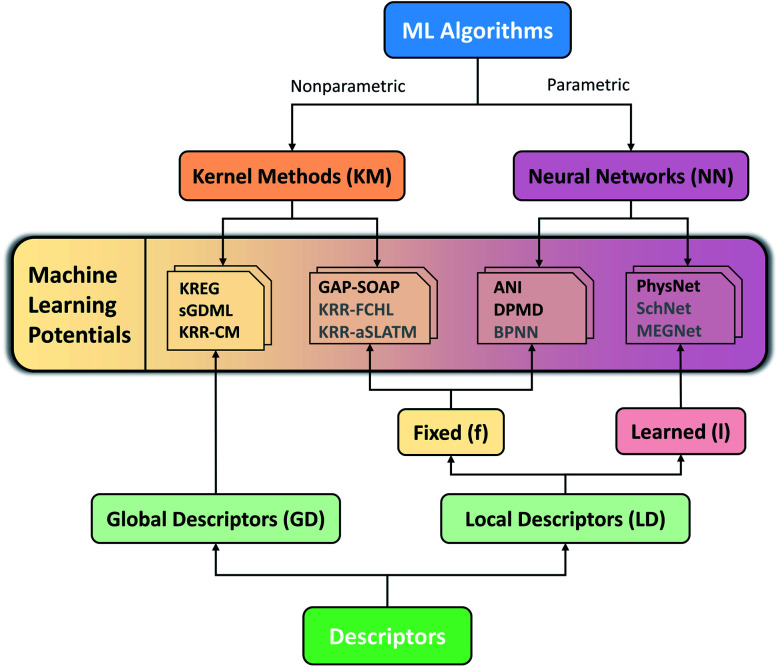
Schematic classification of popular molecular machine learning potentials (MLPs). Representative models on the scheme (models tested here in bold): (1) kernel methods with global descriptor (KM-GD: KREG,^18^ KRR-CM,^41^ and sGDML^20^), (2) kernel method with fixed local descriptor (KM-fLD: GAP^42^-SOAP,^43^ KRR-FCHL,^44^ and KRR-aSLATM^45^), (3) neural networks with fixed local descriptor (NN-fLD: ANI,^46^ DPMD,^47^ and BPNN^48^), (4) neural networks with learned local descriptor (NN-lLD: PhysNet,^36^ SchNet,^49,50^ and MEGNet^51^).

Considering the challenges mentioned above, we concentrate our effort on proposing and testing clear protocols that can guide future benchmark studies on newly published MLPs. These protocols mainly focus on comparing MLPs based on analysis of learning curves on the same data sets, using the same conventions for counting the number of training points by, *e.g.*, including validation points for hyperparameter tuning, ensuring reproducibility of reported results by providing open access to software, sample input files, and output data for learning curves, using the same hardware architecture for comparing timings and other variables. In this sense, the present work should be understood as the first milestone in an open project planned to be constantly updated, and other researchers are welcome to contribute. Our vision is to have an open-access platform, available on http://MLatom.com/MLPbenchmark1, collecting up-to-date comparisons between different MLPs on equal grounds. We encourage the use of protocols outlined in this work and the benchmarks reported here to test any new molecular MLP. As a starting point towards the goal of the open project, we restrict our tests to at least one representative popular MLP model out of four typical combinations of ML algorithm and descriptor (Fig. 1): (1) kernel method with a global descriptor (KM-GD), (2) kernel method with a fixed local descriptor (KM-fLD), (3) neural network with a fixed local descriptor (NN-fLD), and (4) and neural network with learned local descriptor (NN-lLD).

Before discussing the performance of the MLP models, we will introduce the chosen models while briefly overviewing the main aspects of these different classes of ML algorithms and descriptors. We refer the reader to ref. 52 describing technical aspects of the MLPs, which we tested here using our software package MLatom.^34,52,53^ This package has either its own native implementations or invokes MLP models *via* popular third-party programs, which we list in section Code availability.

## Descriptors

### Global descriptors

The descriptor is a numerical representation (usually in a vector form) of a 3D molecular geometry used as input to the ML algorithm. One of the most obvious and simplest descriptors is the Cartesian (*XYZ*) coordinates traditionally used in computational chemistry. It can be classified as a global descriptor (GD) because it describes the entire molecule. *XYZ* coordinates were successfully applied for geometry optimizations accelerated by MLPs.^12,54^ However, such a descriptor does not guarantee by itself compliance with physical constraints, *e.g.*, that rotation and translation of an isolated molecule do not change the potential energy. Other global descriptors used in MLPs^15,17,40,55–57^ are internal coordinates, which are also traditional molecular representations in computational chemistry. They satisfy rotational and translational invariance, but their construction is not unique. This handicap motivated the development and use of specialized global descriptors for MLPs such as the Coulomb matrix (CM),^41^ inverse internuclear distances (IDs),^58,59^ their version normalized relative to equilibrium structure (RE descriptor),^18^ bag-of-bonds (BoB),^60^ and BAML (bonds, angles machine learning).^61^

However, many of the above global descriptors (CM, ID, RE) are not invariant with respect to the permutation of chemically equivalent atoms. Addressing this problem is rather challenging. Some approaches (such as BoB) sort atoms making the representation permutationally invariant. Nevertheless, sorting atoms should be done with care. In many situations (*e.g.*, when using CM, ID, and RE), it may cause PES discontinuities and low accuracy because small geometry changes may lead to drastic changes in the descriptor.^52^ Another solution is summing up the terms arising from internuclear distances for each distinct atom pair as in encoded bonds^62^ (similarly to the approaches adopted in LDs). However, this approach leads to information loss as the structure cannot be uniquely reconstructed from such a descriptor.^62^ A more rigorous solution is to use the permutationally invariant polynomials^63,64^ or fundamental invariants^65^ as descriptors. Nevertheless, due to the quickly growing number of permutations with system size, they can only be applied to small molecules. Other solutions go beyond the descriptor itself. One can learn permutational invariance by expanding the training set with randomly sorted atoms^66^ or modifying the ML algorithm. This latter approach is adopted in permutationally invariant KREG (pKREG)^18,34,52^ and related approaches such as sGDML^20^ and RKHS + F (reproducing kernel Hilbert space using energies and forces),^67^ which use permutationally invariant kernel functions.

Finally, the global descriptors' dimensionality grows with the number of atoms. Although this is not an issue for the PES of small systems like those we discuss here, it may be a problem when dealing with chemically diverse datasets. Thus, special solutions with global representations have been suggested.^62,68,69^

### Local descriptors

Alternatively to global descriptors, one can construct a suitable vector representation for MLPs based on an atom-centered approach. Such a molecular representation is designed to encode the local chemical environment around each atom through a set of basis functions that vanish outside of a cutoff sphere. These representations are called local descriptors (LDs).

Following the concept of many-body expansion, MLPs with local descriptors model the interatomic interactions by decomposing them into *n*-body terms, typically expressed as a combination of radial and angular distribution functions. The advantages of LDs are that they are permutationally invariant and size-extensive by construction and can achieve linear scaling.^43,48^ The list of developed LDs for molecules is extensive. It includes, among others, BP-ACSFs (Behler–Parrinello's atom-centered symmetry functions)^70^ and its ANI-AEV (atomic environment vectors)^46^ and wACSF (weighted ACSF) modifications,^71^ SOAP (smooth overlap of atomic positions),^43^ aSLATM (atomic spectrum of London and Axilrod–Teller–Muto),^45^ FCHL (Faber–Christensen–Huang–Lilienfeld),^44^ Gaussian moments,^72^ spherical Bessel functions,^73,74^ and descriptors used in DPMD (deep potential molecular dynamics)^47^ and DeepPot-SE (DPMD-smooth edition).^75^

Local descriptors can be fixed before training an MLP. Alternatively, the ML algorithm can learn them as a part of the training process (discussed below in the context of NNs). To differentiate these two cases, we denote the first type as fixed LDs (fLDs) and the second type as learned LDs (lLDs).

### Global *vs.* local descriptors

Typically, an MLP using a local descriptor introduces an approximated total energy partitioning into contributions of each atom, whose environment is described only within a predefined cutoff radius. For a single molecule PES, this approximation may be considered conceptually inferior to direct learning of total energies with MLPs using global descriptors. Moreover, many global descriptors are complete descriptors from which the molecular geometry can be reconstructed unambiguously, while the reconstruction is a challenging issue for local descriptors, which can lead to accuracy loss.^76^ However, one should remember that the models based on local descriptors were developed with the motivation to make accurate predictions for larger systems even when trained only on smaller systems rather than solely apply such models to a single molecule PES. Thus, while the elements of global descriptors are often based only on the internuclear distances, local descriptors are usually augmented with additional structural parameters such as angles, which take into account many-body environmental factors to achieve the required accuracy. From a practical perspective, training global descriptors is also more straightforward as they typically do not have extra tunable parameters besides those already existing in the ML algorithms. Local descriptors have additional hyperparameters controlling the cutoff, shape, and size of the basis functions. They are usually system-dependent, and it is recommended to fine-tune them to improve the performance of the final MLP model.^52,77^

Although global and local descriptors are conceptually different, a local descriptor effectively becomes a global one if no cutoff is used. Another way of constructing a global version of a local descriptor is simply by taking the average over all environments.^78^ In this sense, the distinction between local and global descriptors is fuzzy.

In principle, we can use the same descriptor with various ML algorithms. For example, CM was successfully used with both kernel methods^41,79^ and NNs.^80^ Thus, several specialized packages such as *DScribe*^81^ and *MolML*^62^ exist for generating structural descriptors from the molecular 3D geometry. However, many of the descriptors mentioned above have been either developed for or are most often used with a specific ML algorithm available in popular packages. For example, CM is typically used with KRR-based models, mainly because early benchmark studies showed that the combination of KRR-CM is superior to the combination of NN-CM.^66^

## Machine learning algorithms

### Neural network potentials

A NN algorithm takes a descriptor X as input and transforms it into an output Y *via* a set of interconnected processing nodes distributed in layers, processing inputs through weights and biases.^82^ The ability of NNs to describe highly complex data comes from the introduction of an activation function^83,84^ on each node, which produces nonlinear outputs. Indeed, the activation function together with the NN architecture (number of nodes and layers) are essential building blocks of the algorithm that need to be chosen *a priori* but can be optimally adjusted for each type of data and prediction task. Additionally, there are other hyperparameters related to the optimization process (such as optimization algorithm, learning rate, and batch size) and regularization terms (such as dropout layers and batch normalization) that affect the performance of NN algorithms. The NN is trained on a set of data points to find optimal weights and biases by minimizing a loss function using some efficient variant of the gradient descent algorithm within the backpropagation scheme.^85^ This is often a computationally-intensive process, especially for deep networks, which is generally expected to require many training points to reduce the risk of overfitting due to the large number of parameters to be optimized. Additionally, a high extra computational cost comes into play if derivatives have to be included in the loss function, as we will see below. However, because of its parametric formulation, the computational cost of evaluating a pre-trained NN algorithm depends mainly on the chosen architecture rather than the amount of data.

A classical example of MLPs based on NNs is the Behler–Parrinello NN (BPNN), which employs BP-ACSFs as a fixed local descriptor. Individual feed-forward NNs are designed and trained for each chemical element in the system so that the sum of atomic contributions approximates the total energy.^48^ This NN-fLD model inspired a progressive and systematic improvement in the architecture and accuracy of NN potentials reported in the literature.^47,50^ A notable example of such advancement is the ANI family of MLPs.^2,46,86^ Many ANI models, trained on a chemically diverse dataset spanning both configurational and conformational spaces, are available and can be used out-of-the-box. A similar concept but with different descriptors is used in DPMD, another successful example of an NN-fLD MLP.^47^ DPMD belongs to the first generation of deep learning models for molecular simulations, which have been continuously improved from both an efficiency^87^ and accuracy^75^ perspective. The descriptors in DPMD are defined in a local coordinate system, which gives some flexibility in generating them.

At a higher level of model complexity, there are deep learning architectures of the “message-passing” type, also known as graph (convolutional) neural networks (GNNs).^88,89^ This approach takes advantage of the inherent graph topology of molecular materials by assigning node vectors in a graph network to each atom. These node vectors, in turn, share information about their local chemical environment in an iterative process *via* updating functions connecting all the nearest nodes. Generally, the initial stage of the training process in GNNs includes an iterative scheme where each representation vector stored in the nodes is updated using the message obtained from the neighboring nodes and the previous state of the modified node. In this way, the GNNs can learn an optimal representation for the system by progressively encoding high-order interatomic relations in a data-driven manner, giving rise to the class of so-called learned local descriptors (NN-lLD models). Examples of such models are DTNN (Deep Tensor Neural Network),^90^ SchNet,^49,50^ PhysNet,^36^ and MEGNet (MatErials Graph Network).^51^

### Kernel method potentials

Kernel methods are a class of learning algorithms that keep a memory of the training examples to predict an output Y for a new input X as a linear combination of similarity basis functions (the so-called kernel functions) centered on each training point.^30^ The linear coefficients used in this expansion are usually determined by solving a convex optimization problem. Thus, the performance of kernel methods is strictly related to the kernel function's ability to capture the similarity between pairs of data points, and designing new kernel functions optimized for molecular systems is a very active research field in computational chemistry.

In contrast to NNs, descriptors are used in a very different way in kernel methods. In these methods, a descriptor for a new geometry enters the kernel function measuring the similarity of this descriptor to each other in the training set.^91^ The learned parameters of a kernel method algorithm are then regression coefficients scaling the kernel functions. Consequently, the number of parameters grows with the training set size and slows down the MLP model's evaluation. On the other hand, these parameters can be found analytically by solving a system of linear equations, turning the fitting process more manageable than in NNs for not too large training sets.^91^ For extensive training sets, special approximation techniques exist to speed up a kernel-method training and evaluation.^91^ Kernel methods have become a popular choice of regression estimator in chemistry thanks to their efficiency in describing highly complex nonlinear functions even for small training sets.^9^ There are many different kernel functions (*e.g.*, the Gaussian, Laplacian, and polynomial kernel functions) for which the prediction performance usually depends on the data representation.^66^ It is also possible to combine a predefined set of kernel functions and learn the optimal (linear or nonlinear) combination as a part of the algorithm in an approach called multiple kernel learning.^92^ Hence, a primary task for kernel methods is to select the best performing kernel function carefully. The choice of kernel function and their parameters together with the regularization parameters are considered hyperparameters of kernel methods.

In the kernel method framework, one of the simplest approaches to perform regression on nonlinear data is KRR, a kernel-based version of the linear ridge regression algorithm. Formally, the learning problem in KRR is expressed in terms of an inversion operation on the regularized kernel matrix instead of minimizing an objective function as commonly done in many others ML algorithms.^93^ This approach has the advantage of providing a closed-form solution^94^ that guarantees that the global minimum is found and, consequently, less prone to over-fitting. On the other hand, the computational cost of kernel methods and the memory size required for storing the kernel matrix rapidly grow with the number of data points (training time scales as *O*(*N*^3^), matrix size as *O*(*N*^2^), and prediction time as *O*(*N*)).^34^ Finding the optimal set of hyperparameters can be cumbersome in KRR due to the lack of an efficient/unique route for searching in the vast hyperparameter space. Such a route is in principle provided (albeit not necessarily exploited) by another kernel method—the Gaussian process regression (GPR),^91^ which has also been intensively exploited in chemistry.^9^

In principle, both KRR and GPR give identical predictions for the same hyperparameters, but GPR is derived from another formalism based on a Bayesian probabilistic model.^91^ It is grounded on the statistical assumption that the data set follows a multivariate Gaussian distribution, specified by a mean function and a covariance kernel function that expresses the similarity between data points as in KRR.^91^ This leads to an important possibility of naturally having in GPR a direct measure for the variance or uncertainty of the predicted quantities by construction.^91^

The most straightforward kernel-method application in MLPs is to directly learn the total energies using global descriptors as is done in the KREG model or a popular combination of KRR with CM (the KRR-CM model). When derivative information needs to be included for training, two distinct approaches have been suggested. One is to solve the overdetermined system of linear equations including derivatives of MLP as in so-called “operator quantum machine learning” with FCHL^44,95^ (analogous to including derivatives in the loss function of NNs). Another one is explicitly including either or both covariances between functions and function derivatives in the system of linear equations^91^ as was done using many potentials, such as GDML,^58^ sGDML,^20,96^ RKHS + F,^67^ GPR with either *XYZ*^12,54^ or internal coordinates,^15,17,57^ FCHL,^44^ and GAP (Gaussian approximation potential).^42^ Explicit inclusion of covariances between function derivatives greatly increases the accuracy of MLPs, but the computational cost rapidly grows as the number of linear equations to solve increases with the number of force components in a molecule.

Many kernel-based potentials also partition the energy into atomic contribution similarly to popular NN potentials. As in NNs, a challenge arises from the absence of the reference atomic contributions because there is no unique way to calculate them using quantum chemistry. Nevertheless, quantum-chemistry approaches based on Bader analysis^97^ have been applied to generate atomic contributions for training kernel methods^98^ and NN^99^ potentials. Alternatively, kernel methods can partition the total energy into atomic contribution during training by solving the correspondingly modified system of linear equations as is done in GAP,^42^ FCHL,^44,95^ and aSLATM.^45^ Kernel-based potentials with local descriptors tend to be more costly than those with global descriptors because they require evaluating the kernel function for many local environments.

## Performance evaluation

Having completed the brief tour in popular types of molecular MLPs, we are now in a position to describe the models chosen for our tests in the context of the MLP classification we introduced (see Fig. 1). Altogether we tested seven MLPs. Since the popular software packages tend to use mainly combinations of global descriptors with kernel methods rather than with NNs, we have chosen the following KM-GD models: (1) KREG (unsorted version, if not mentioned otherwise), (2) sGDML, and (3) KRR with unsorted CM and Gaussian kernel function (KRR-CM). To represent KM-fLD models, we have chosen (4) GAP-SOAP. Among NN methods, NN-fLD is represented by an (5) ANI (NN with ANI-AEV descriptors) and (6) DPMD models. Finally, we have taken the (7) PhysNet model as representative of NN-lLDs. For all kernel-based models, we optimized hyperparameters using default settings as described elsewhere.^52^ Due to the high cost of training the NN-based models, we did not optimize their hyperparameters.

Given the plethora of available MLPs and the different technical details related to their implementation and application, it is rather challenging to assess their performance systematically and on equal footing. Although the accuracy of these MLPs has been reported for different datasets, the data types (configurational, compositional) and the size of the training and validation sets are often not the same, hindering a direct comparison between results. Furthermore, it is insufficient to compare just models trained on the same, specific number of points. An essential aspect that must be considered is how the models' accuracy changes with the training set size, *i.e.*, one should compare the learning curves^100^ of different models. To facilitate comparing different MLP model performances, we have recently extended the MLatom package with a modularized Python interface to popular third-party ML programs. The package offers researchers a unified platform with a standardized input/output structure to perform ML simulations for atomistic systems.^52^ This integrated platform allows for automatic generation of the learning curves for errors in energies and forces and their standard deviations. It also allows for hyperparameter optimization and recording performance metrics such as the training and prediction times and their standard deviations. All the numerical results reported here are openly available together with the sample MLatom input files on DOI: 10.6084/m9.figshare.c.2878631.

We adopted the extended version of the popular MD17 benchmark database^20,58^ to evaluate the performance of the chosen MLP models because molecular dynamics is one of the main applications of MLPs. This database is composed of independent configurational datasets generated through quantum-chemical molecular dynamics (with *T* = 500 K) for ten organic molecules with sizes ranging from 9 (ethanol and malonaldehyde) to 24 atoms (azobenzene). Thus, this database also allows us to investigate how molecular complexity influences the ML model's performance. As target quantities for predictions, the MD17 database includes potential energies and forces determined with van der Waals-corrected density functional theory with the PBE functional (DFT/PBE + vdW-TS).^101,102^

In the following, we evaluate the performance of MLPs trained either only on energies or simultaneously on energies and forces considering all MD17 datasets.

### Training PES on energies only

**Fig. 2:**
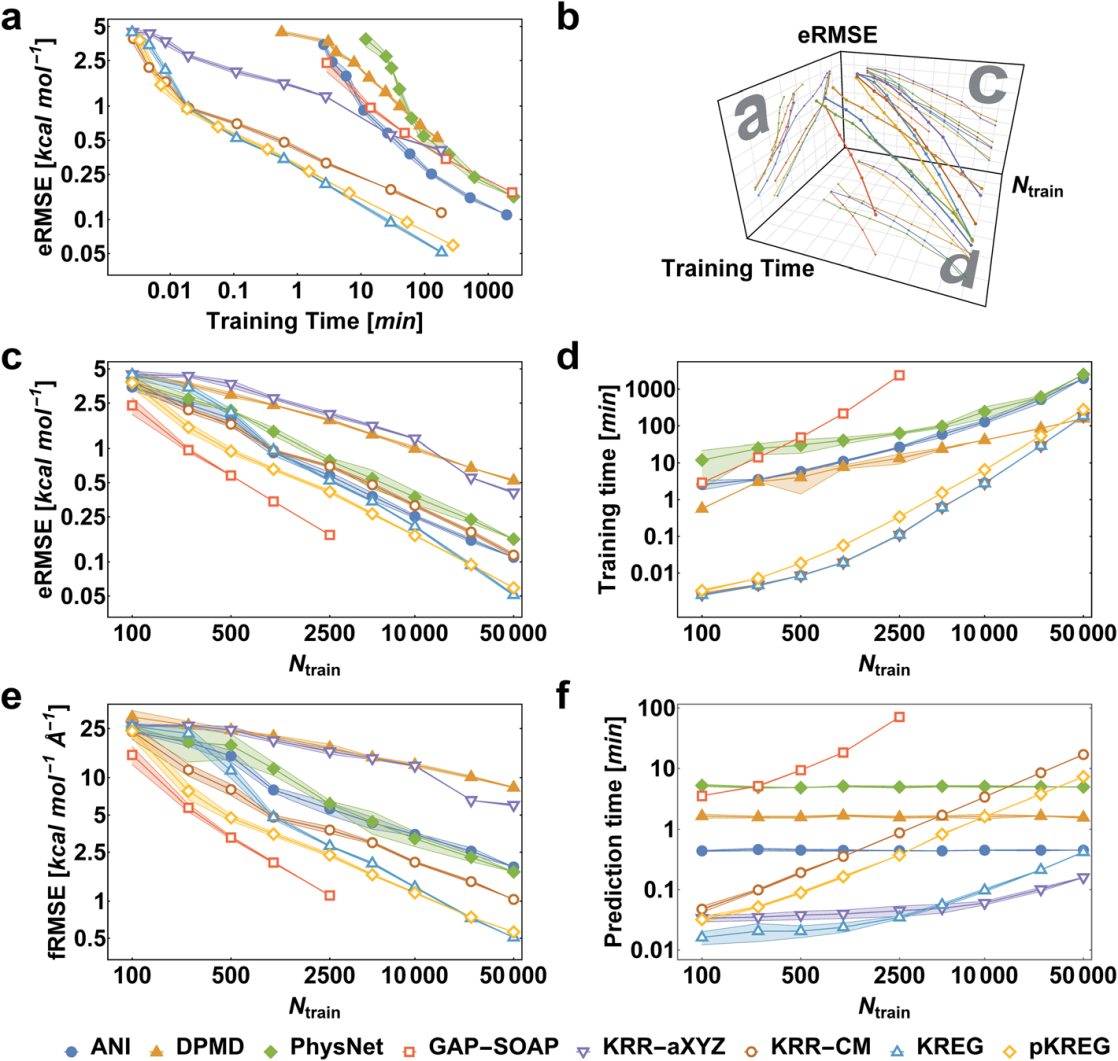
Performance curves of the different machine learning potentials trained only on energies for the MD17-ethanol dataset. (a) Root-mean-squared error in energies (eRMSE) *vs.* training time. (b) Plot of models' performance in the 3D space of the number of training points (*N*_train_), training time, and eRMSE. (c) eRMSE *vs.* the number of training points. (d) Training time *vs.* the number of training points. (e) RMSE in forces (fRMSE) *vs.* the number of training points. (f) Time for predicting energies and forces for 20k points in the test set *vs.* the number of training points. All computations were performed on two processors with 36 Intel Xeon(R) Gold 6240 CPUs (2.60 GHz). The shaded areas in the plots correspond to one standard deviation. Filled markers are used for neural network potentials, while empty markers for kernel method potentials.

We first consider the performance for the simplest case, training MLPs only on energies. This approach is often necessary when no PES gradient information is readily available at the quantum-chemical level. As an illustrative example, we show the performance curves for ethanol in Fig. 2. (Note that sGDML cannot be trained only on energies, thus not being evaluated for this task.) For large enough training sets, all models can achieve remarkably high accuracy for predicting energy (root-mean-squared error [RMSE] below 1 kcal mol^−1^, corresponding to the so-called “chemical accuracy” desired for quantum-chemistry applications). Nevertheless, only the kernel-based potentials GAP-SOAP, KREG, and KRR-CM could achieve RMSE for forces close to or below 1 kcal mol^−1^ Å^−1^ for the considered range of training set sizes. Learning curves for both energies (Fig. 2c) and forces (Fig. 2e) follow similar trends. On the other hand, the somewhat surprisingly poor performance of DPMD shows that even relatively advanced MLPs not adequately tuned for a particular application do not by itself guarantee better accuracy than much simpler models such as KRR with aligned *XYZ* (KRR-a*XYZ*), which is used as a baseline model for a sanity check.

This point becomes particularly relevant if one considers that KM-GD models (KRR-a*XYZ*, KREG, and KRR-CM) show a much shorter training time compared to any other model almost for the entire range of training set sizes investigated here (Fig. 2d). As a result, these KM-GD potentials can achieve the same accuracy as other models within a much shorter training time (Fig. 2a). For the vast training sets (more than 50k points), bad scaling of KM training and rapidly increasing memory required to store the kernel matrix is expected to make training of KM potentials infeasible.^34^ Thus, training with NN or implementing sparsification techniques for KM-GD potentials would be necessary for vast training sets. As a side note, one could in principle reach a better efficiency in the training of both NN and kernel methods working on GPU (Graphical Processing Unit) hardware (although less well-known, in the case of kernel models, specialized software programs have been developed to enable such an efficiency-boosting too).^103,104^ However, for comparing timings on the ethanol data set, we performed all simulations on CPUs, using the same computer hardware architecture and number of cores to allow for a fair comparison between different models, alleviating possible dependencies of the computational timings concerning specific hardware configuration.

When using MLPs, we should also consider their performance for making predictions as it can be a decisive factor in enabling massive computations like *e.g.*, very long molecular dynamics. The prediction times for KM-GDs models such as KREG and KRR-a*XYZ* trained with up to 50k points are again much lower than for other models (Fig. 2f). The prediction time with NNs does not depend on the number of training points as expected from its parametric definition.

GAP-SOAP delivers the best accuracy for ethanol (but not for all other molecules; see below). Nevertheless, its particularly bad training-time and prediction-time scaling with the increasing number of training points makes it quickly computationally intractable (Fig. 2d and f). One can speed up GAP-SOAP using approximation techniques (applying smaller cutoffs and sparsification), but at the expense of accuracy loss, so one may be better off opting for faster KM-GDs approaches. Conversely, KM-GDs and NN approaches can also be made more accurate by modifying the model, often at an increased computational cost. One example is our pKREG model. Compared to unsorted KREG (called simply KREG), the inclusion of permutational invariance as additional physical information significantly improves the accuracy for small and moderate-size training sets (up to *ca.* 10k points) while only slightly increasing the training time (Fig. 2c and d).

**Fig. 3:**
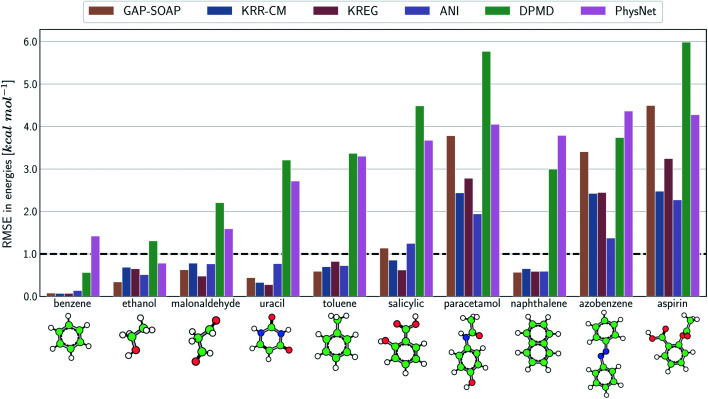
Performance of MLPs trained only on energies. Root-mean squared error (RMSE) calculated for energies with different ML potentials for all molecules of the MD17 database. The models were trained on a sub-sample of 1000 molecular geometries for each system. The reported RMSE values correspond to the average of the test errors for 20 independently trained models evaluated on a test set with 20k geometries.

The same general conclusions—drawn here for ethanol—also hold for other molecules. However, keeping track of the learning performance of MLPs across the compositional chemical space (*i.e.*, considering different molecules) becomes more challenging since we add one more dimension to the learning curve analysis. One possible way to analyze the model's accuracy for the whole MD17 database is to look at a slice through the learning curves at 1000 points, focusing on how the different models perform with increasing molecular complexity (Fig. 3). All models decrease their accuracy for larger and more flexible molecules, requiring more training points to achieve the desired accuracy (results for other training-set sizes are available at DOI: 10.6084/m9.figshare.c.2878631). Nevertheless, the impact of complexity is different for different models, and, *e.g.*, GAP-SOAP is no longer the best model for salicylic acid (where KREG is the best) and aspirin (where ANI and KRR-CM are better). Other examples are DPMD, which is more accurate than PhysNet for half of the molecules, and ANI, which has accuracy comparable to that of kernel methods in many cases.

**Fig. 4:**
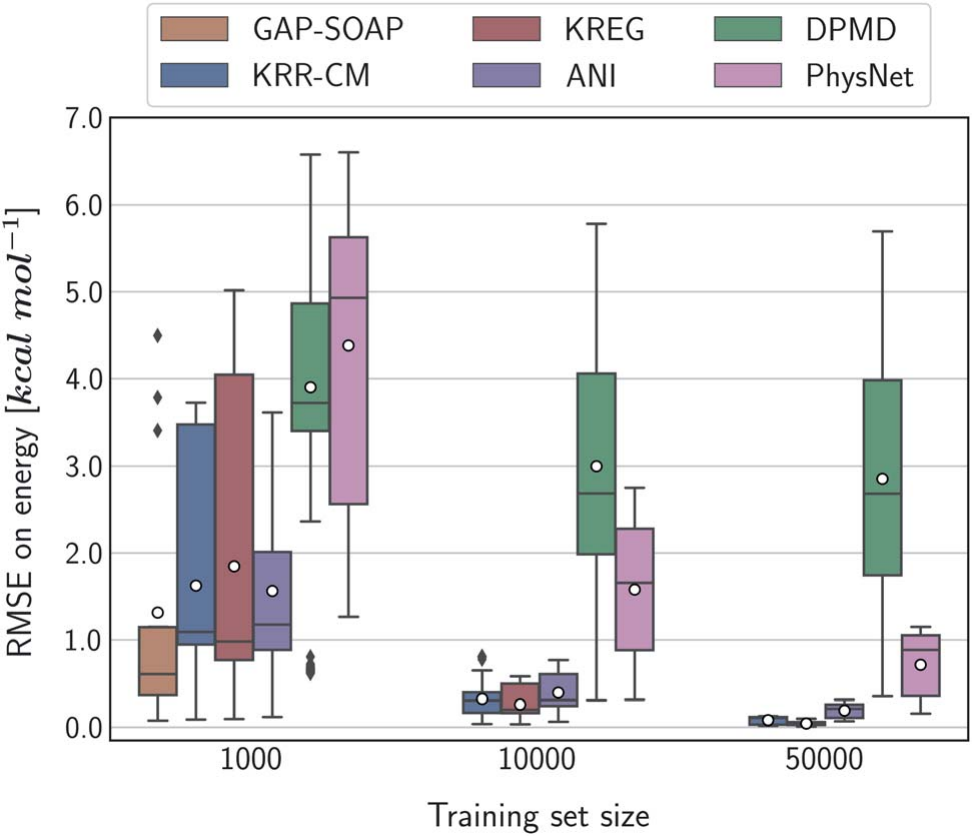
Box plot representation of the MLPs' learning performance across the compositional space of the MD17 database. All ML models were trained only on energies. Each box plot represents the distribution of the root-mean-squared error (RMSE) for the total energies of all molecules in the MD17 database calculated with respect to the true labels of a test set with 20k geometries. The white dots in the box plots correspond to the mean value of the RMSE for all different molecules.

Alternatively, an overall picture of the learning performance of different MLPs can also be obtained by representing the (sparse) learning curves as box plots^105^ characterizing the distribution of the RMSE values for all molecules of MD17 database (Fig. 4). The wide error bars obtained for models trained on 1000 points clearly demonstrate the dependence of the MLP's performance with respect to the molecular complexity. Although GAP-SOAP shows the smallest error bar for 1000 training points, the RMSE of energy predictions for azobenzene, paracetamol, and aspirin (3.4, 3.8, and 4.5 kcal mol^−1^, respectively) appears as outliers in the box plot, suggesting that the model's hyperparameters or the SOAP descriptor might not be well suited for these molecules. The size of the error bars quickly decreases for most MLPs (except for DPMD) when the training set is augmented by adding more geometries. In terms of accuracy, the tiny error bars (varying below 1 kcal mol^−1^ for 10k training points) obtained for both kernel methods, KRR-CM and KREG, and the NN model ANI indicate that these models are competitive in consistently predicting the total energies with high accuracy for the different molecules of the MD17 database when only energies are available for training.

### Training PES on energies and forces

**Fig. 5:**
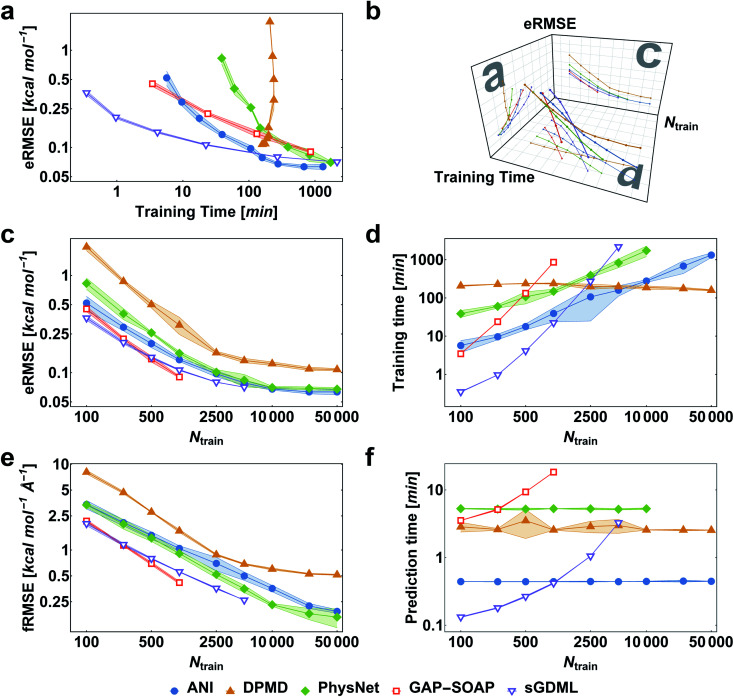
Performance curves of the different machine learning potentials trained on energies and forces for the MD17-ethanol dataset. (a) Root-mean-squared error in energies (eRMSE) *vs.* training time. (b) Plot of models' performance in the 3D space of the number of training points (*N*_train_), training time, and eRMSE. (c) eRMSE in energies *vs.* the number of training points. (d) Training time *vs.* the number of training points. (e) RMSE in forces (fRMSE) *vs.*, the number of training points. (f) Time for predicting energies and forces for 20k points in the test set *vs.* number of training points. All computations were performed on two processors with 36 Intel Xeon(R) Gold 6240 CPUs (2.60 GHz). The shaded areas in the plots correspond to one standard deviation. Filled markers are used for neural network potentials, while empty markers for kernel method potentials.

When reference forces are available, one can include this information in the training to improve the MLP accuracy.^43,48^ Indeed, our tests also clearly confirm a significant reduction in prediction error for all MLPs (Fig. 5). Learning curves for MLPs evaluated on the ethanol dataset are shifted to higher errors for models trained only on energies (Fig. 2) in comparison to those trained on energies and forces (Fig. 5). Note that KREG, KRR-CM, and KRR-a*XYZ* are not evaluated because learning on energies and forces is not implemented for these models.

**Fig. 6:**
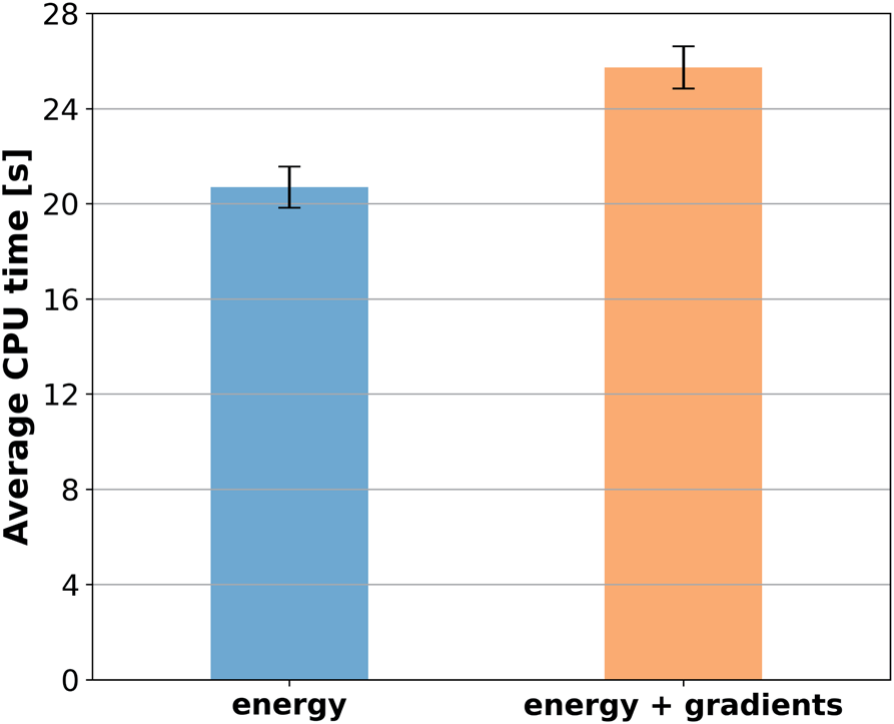
Computational cost for generating training sets for toluene with DFT. The bars represent the average CPU time required to perform 1000 single-point calculations where either only total energy or energy plus forces were computed. The calculations were performed with the Orca program using the PBE0 functional, the 6-311G(d) basis set, and very tight settings for the SCF convergence.

However, to answer whether it is worth including forces in the training set, we must consider the additional computational cost due to the quantum-chemistry calculations of forces and the substantially longer training time. The extra time required for calculating quantum-chemical forces is usually small for standard DFT implementations (Fig. 6), but it may be much larger for other methods. For instance, the cost of calculating forces with CCSDT(Q) (a state-of-the-art quantum-chemistry method) is between two to three times the cost of the energy calculation.^106^

Compared to training on energies and forces, our analysis shows that to achieve the same target accuracy when training only on energies, one needs substantially more data points to compensate for the lack of information about the PES curvature (given by the forces). For example, if we need an RMSE in forces of *ca.* 1 kcal mol^−1^ Å^−1^ for ethanol, we would need to train GAP-SOAP on 2500 energy points, but only on 250 energy + force points. With respective training times of 40 against 4 hours, even an overhead factor of 8 in the force calculation times would still favor training on energy + force. Another example, the NN potentials ANI and PhysNet can easily achieve 1 kcal mol^−1^ Å^−1^ with *ca.* 1000 training points with forces. However, as discussed above, even 50k training points without forces are not enough to reach such an accuracy. Thus, the inclusion of forces is justified and recommended in all cases where they are available.

Among the various MLPs tested here, the KM-GD model sGDML requires much less training time to achieve a remarkably lower error than other approaches for small training set sizes (up to *ca.* 1k points, Fig. 5a and d). For larger training sets, the training time and memory requirements of kernel-based potentials become too high. Then, one can either use approximation techniques such as sparsification or simply use NN potentials, which become a convenient choice in this scenario. Among the latter, NN-fLD potentials are generally more computationally efficient than NN-lLD potentials. For example, the ANI training and prediction times are about factor 10 shorter than those of PhysNet (Fig. 5d and f, respectively).

**Fig. 7:**
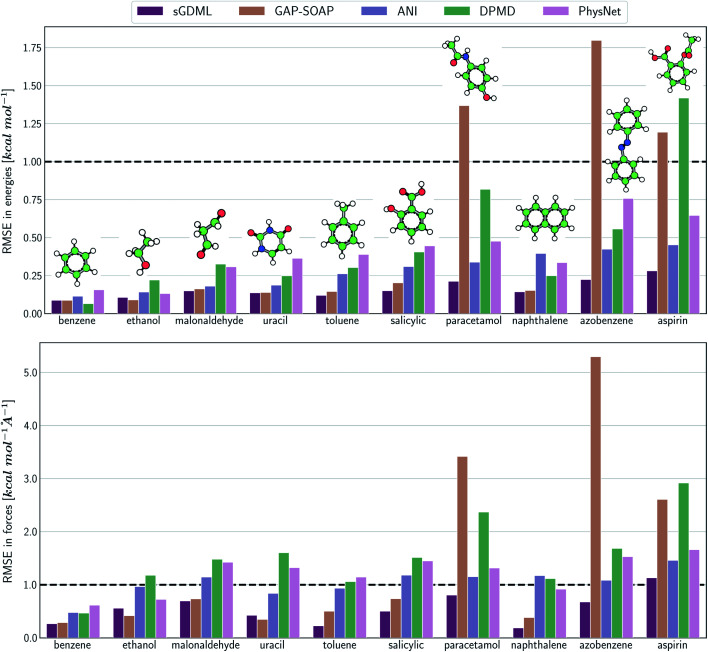
Performance of MLPs trained on energies and forces. Root-mean squared error (RMSE) calculated for energies (top panel) and forces (bottom) with different ML potentials for all molecules of the MD17 database. The models were trained on a sub-sample of 1000 molecular geometries for each system. The reported RMSE values correspond to the average of the test errors for 20 independently trained models evaluated on a test set with 20k geometries.

**Fig. 8:**
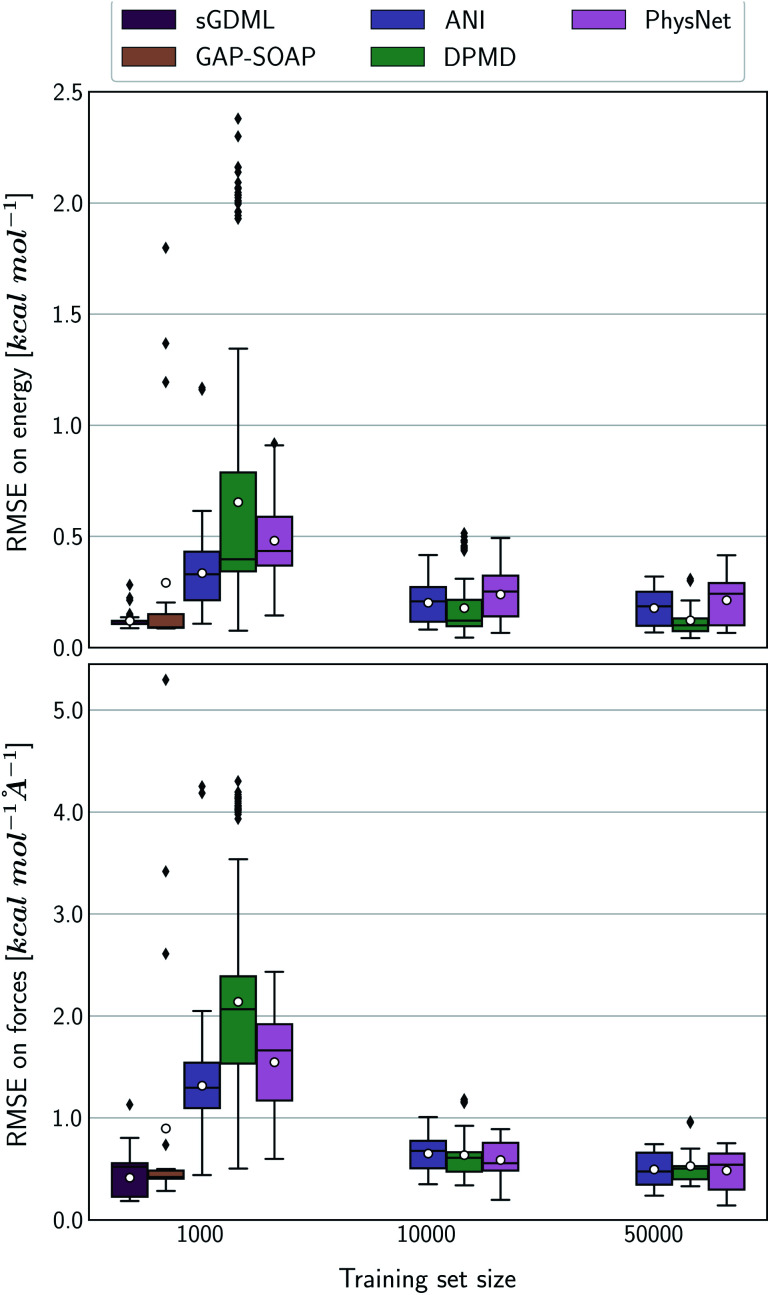
Box plot representation of the MLPs' learning performance across the compositional space of MD17 database. All ML models were trained using energies and forces information. Each box plot represents the distribution of the root-mean squared error (RMSE) for the total energies of all molecules in the MD17 database calculated with respect to the true labels of a test set consisting of 20 000 geometries. The mean value of RMSE calculated for all different molecules is represented as white dots, while black markers represent outliers.

The accuracy of the NN-based potentials strongly depends on the system. For example, while DPMD and ANI are better for benzene, PhysNet is much better for ethanol (the slice through learning curves at 1000 points is shown in Fig. 7; results for other training sets are available at DOI: 10.6084/m9.figshare.c.2878631). Such dependencies in the ML model's performance regarding molecular complexity can also be inferred from the box plots in Fig. 8. While kernel methods (sGDML and GAP-SOAP) show a narrow and well-converged distribution of RMSE values (with most of the errors lying below 1 kcal mol^−1^) already for a small training set size (1000 points), the NN models need much larger training sets to achieve the same level of accuracy. Again, GAP-SOAP shows poor performance for aspirin, paracetamol, and azobenzene (see outliers in Fig. 8) even when forces are used in the training. In DPMD, the worst energy and force predictions, corresponding to the outliers in the box plots, are always obtained for aspirin no matter the size of the training set.

All in all, ANI is consistently robust in terms of accuracy across different molecules, which, combined with its excellent computational efficiency, makes it a good default choice for large training sets. We should also note that the weight assigned to forces in the loss function varies in different models,^36,46,47^ which means that there is potential to improve ANI for forces even more. On the other hand, DPMD only shows good performance in terms of both accuracy and computational efficiency for rather large training sets with more than 10k points (Fig. 5d and 8; note that the training time of DPMD is roughly constant).

## Training set sampling and conformational analysis

Another critical point is that the accuracy of machine learning can quickly achieve lower error than the inherent error of the typical quantum-chemistry approaches used to generate the training data. Thus, in many cases, we can expect that simulations with different MLPs would lead to similar results, but to what extent the error in ML influences the final result is yet to be explored.

Finally, maybe the most critical question in MLP model development is not improving the models themselves but the efficient generation of good training data, which is, therefore, a topic of intensive ongoing research.^9^ These data should be balanced because significant undersampling of a region in the conformational space may lead to a drastic drop of accuracy for this region, introducing a bias in the ML potential and, as a result, quantitatively wrong conclusions from simulations with such a potential.

**Fig. 9:**
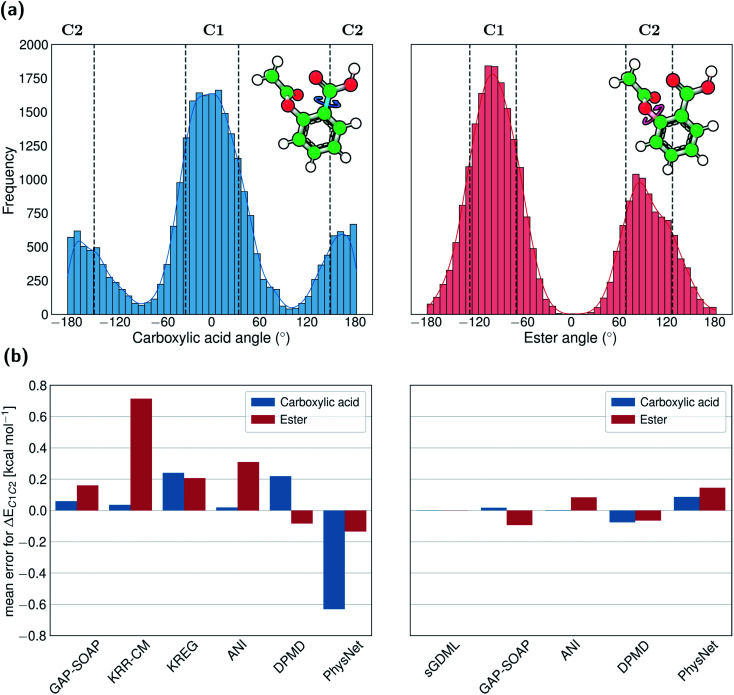
Performance of MLPs for conformational energies of aspirin. (a) Histogram of the dihedral angles distribution describing the most frequent conformations found in the full MD17-aspirin data set with respect to the rotation of the carboxylic acid (left panel) and ester (right panel) groups. Vertical lines are drawn to indicate the threshold of dihedral angles (mean ± standard deviation) used to select clusters of conformers around the local minima. (b) Errors of MLPs trained with 1000 points on energies (left panel) and using energies and forces (right panel) in predicting the difference between the mean energy of conformer clusters C1 and C2 (data at https://figshare.com/s/6cf4347e2682829bff75). For the carboxylic group, the difference between the mean energy of conformers C2 and C1 is 0.87 kcal mol^−1^, while this difference is only 0.02 kcal mol^−1^ for the ester group.

For example, aspirin has many different conformational isomers, and training data sampled from molecular dynamics will probe each of them differently. If we focus on the two conformations formed by rotation around the Ph–C bond of the carboxylic acid, the ratio between points populating them in the entire MD17 dataset is 1 : 3. On the other hand, if we focus on the two conformations arising from rotations around the Ph–O bond of the ester, the population ratio is 1 : 2 (Fig. 9a).

The disbalance in the distribution of training data for each conformation cluster results in systematic errors in MLP predictions indicated by rather significant errors in the mean energies of one cluster relative to another (up to 0.8 kcal mol^−1^ for KRR-CM, Fig. 9b). The only exception is an excellent performance of sGDML, meaning that using overall more accurate ML potential may be necessary if only a small amount of training data in regions of interest is available. These findings highlight the importance of performing a more homogeneous sampling of the configurational space to construct high-quality training sets. Indeed, as detailed in a recent publication,^107^ the predictive performance of various ML potentials can be significantly boosted by applying unsupervised learning techniques to control the undersampling of physically relevant molecular structures that fall into low probability regions of the configurational space.

## Conclusions and outlook

In the last years, many different ML potentials to predict the potential energy of molecules have been developed. Amidst the profusion of acronyms, it's becoming a formidable challenge for the non-specialist (and sometimes even to specialists) to understand the differences between these ML potentials and evaluate how good each one is for a specific application. In this work, we have shown how to recognize the main features of each ML potential in terms of ML descriptor and algorithm, surveying their main types and their main pros and cons.

Moreover, we have critically analyzed a wide range of molecular ML potentials based on kernel methods and neural networks combined with global and local descriptors from the standpoint of accuracy and computational efficiency. Our goal has been to provide qualified information to aid researchers in choosing the most suitable potential for their applications.

When only energies and no forces are available for training, ML potentials based on kernel methods with global descriptors such as KREG and KRR-CM should be favored because they are both more efficient and accurate than other approaches. For small training sets (up to *ca.* 1000 points), kernel methods with local descriptors such as GAP-SOAP can also be considered for use as they often have excellent accuracy. For vast training sets (larger than 50k points—the largest training set investigated here), neural network potentials are expected to be preferred due to their better computational scaling and lower memory requirements.

If reference forces are also available, they should be included in the training set. This procedure substantially improves the accuracy, and the computational overhead is practically always justified. When training with energies and forces, kernel methods with global descriptors such as sGDML are recommended for small training sets (up to *ca.* 1k points). They provide the best trade-off between accuracy and computational cost. For large training sets, neural network potentials become a better choice. Among NN models, potentials with fixed local descriptors such as ANI and DPMD allow better performance and, in many cases, also provide better accuracy. However, in some cases, potentials with learned local descriptors such as PhysNet may be more accurate (*e.g.*, it has smaller errors for forces in ethanol, Fig. 5e).

**Fig. 10:**
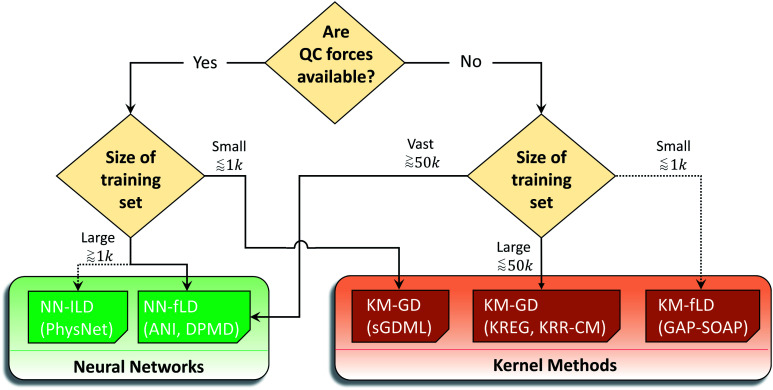
Flowchart for a data-oriented selection of the “right” machine learning potential in terms of both accuracy and efficiency. The proposed flowchart considers the trade-off between accuracy and computational cost related to the application of potentials in different scenarios. The solid lines indicate the generally preferable potentials, while dashed lines indicate secondary options that could still be explored in more specific applications.

We summarized these recommendations in the flowchart given in Fig. 10. Naturally, these recommendations, even supported by the data, may be biased by the type of research we do. Thus, the researcher choosing an MLP may follow them for a quick qualitative assessment, but they should benchmark MLPs in the view of their own applications as it is common while choosing a quantum-chemical method.

Although kernel methods tend to outperform NNs in small training-set scenarios when trained on energies and forces, the computational overhead for training a kernel-based potential may become an issue in applications requiring many data points. Such would be the case of constructing PES for isomeric molecular datasets (*e.g.*, ISO17 dataset^49^), describing complex dynamical reaction processes,^26^ or fitting PESs in the full chemical (compositional and configurational) space with a single MLP model. Therefore, a detailed benchmark study to elucidate these issues would close a critical gap in the literature by providing helpful, evidence-based, and numerically-supported guidelines for selecting and developing efficient ML potentials.

The future development of MLPs may focus on the implementation of sparsification techniques, *e.g.*, by including forces information only for critical regions such as near transition state structures. It is worth pursuing to make kernel methods with global descriptors more efficient, especially when trained on energies and forces. In addition, it is desirable to improve the accuracy of kernel methods with global descriptors, mainly for energy-only training, as they provide cost-efficient training but may be less accurate than kernel methods with local descriptors.

While in the future it is certainly worth performing extensive comparative tests of the applicability of diverse MLPs to practical simulations ranging from molecular dynamics to spectroscopy, generally speaking, the field of MLPs is rapidly maturing, and it is time to focus the effort to improve the computational performance of existing models, *e.g.*, by extensively optimizing software packages. Such efforts are underway in many groups, with one striking example of using MLPs for simulating 100 million atoms.^87^

Disbalance in conformational samplings of the training set data is a critical problem for the accuracy of MLPs. We have seen that for small training sets, expensive MLPs may be required to mitigate the problem. Moreover, using and developing guided sampling approaches, *e.g.* active learning based on molecular dynamics,^19^ metadynamics,^108^ or global optimization algorithms such as stochastic surface walking-neural network approach,^13^ should be the focus of the future investigations.

In conclusion, the detailed computational protocols for training and testing machine learning models, the unified software infrastructure, online open-access platform collecting comparisons between different MLPs and provided herein (available on http://MLatom.com/MLPbenchmark1) lay the groundwork for bridging the gap between the development of new MLPs and testing their performance on equal footing.

## Methods

All machine learning potentials were trained with default settings of MLatom 2.0.3 as described in ref. 52. The GAP-SOAP model was trained without cutoff and sparsification to avoid arbitrariness in choosing these hyperparameters. The first geometry in each data set was used as the reference geometry for calculating the RE descriptor in the KREG models.

In the case of the neural network models (ANI, DPMD and PhysNet), the ethanol dataset was used as the basis to calibrate hyperparameters when the values were not available in the original publication. Once we have found the hyperparameter settings that better reproduce the testing error reported in the literature, we use the same settings for the remaining molecules of the MD17 database. These hyperparameters are used as the default in the MLatom interface module. Therefore, further improvements in the NN model predictions provided in this work might be achieved by fine-tuning the hyperparameters to each specific dataset. The early stopping method was used in the training process to prevent overfitting, either in its original implementation when available (TorchANI) or through our own implementation in the MLatom interface with other packages (DeePMD-kit and PhysNet).

To generate a*XYZ* descriptors, first, we defined the coordinate origin at the molecule's centroid. Then, the Kabsch algorithm^109^ was applied to align their orientations with respect to the first geometry of the reduced data set with 20k test points of each molecular PES. Finally, the aligned geometries were flattened to 1D arrays as the X input of MLatom. No weights for different atoms were used in the alignment.

The DFT single-point calculations (PBE0/6-311G*) were performed with ORCA.^110^ The energies and forces were calculated using a dense integration grid (given by the Grid5 and NoFinalGrid keywords, corresponding to 434 points in the Lebedev grid), and also using a very tight criteria for the self-consistent field (SCF) as given by the VeryTightSCF keyword (tolerance of 10^−9^ hartrees for energy change and 2.0 × 10^−6^ for orbital gradient rotation angle convergence).

## Data availability

The data that supports the findings of this study are available from DOI: 10.6084/m9.figshare.c.2878631.

## Code availability

MLatom package is available free of charge for non-commercial and non-profit uses at http://MLatom.com. MLatom with the settings used to perform the simulations in this study is available as an open-source code and executables on the aforementioned website and can be also installed using the command pip install MLatom==2.0.3. List of programs interfaced in MLatom and their versions used in this work are provided in Table 1. Scripts for aligning *XYZ* (a*XYZ* descriptor) and forces are available at http://MLatom.com/MLPbenchmark1 and DOI: 10.6084/m9.figshare.c.2878631. The code with the Kabsch algorithm is available as a part of the program for calculating root-mean-square deviation between two molecular geometries at https://github.com/charnley/rmsd.

**Table 1:** List of programs interfaced in MLatom and used in this work

ML potential	Program	Version	Repository
ANI	TorchANI	2.2	https://github.com/aiqm/torchani
DPMD	DeePMD-kit	1.2.2	https://github.com/deepmodeling/deepmd-kit
PhysNet	PhysNet	e243e2c	https://github.com/MMunibas/PhysNet
sGDML	sGDML	0.4.4	https://sgdml.org
GAP-SOAP	GAP	1598976566	https://www.libatoms.org/gap/gap_download.html
	QUIP	5c61598e4	https://github.com/libAtoms/QUIP

## Author contributions

P. O. D. and M. B.: conceptualization, supervision, and funding acquisition. P. O. D.: project administration. P. O. D., M. P. J., and F. G.: software. N. F. and P. O. D.: resources. P. O. D. and F. G.: methodology and validation. M. P. J. and F. G.: investigation, visualization, data curation, and formal analysis. M. P. J. and P. O. D.: writing – original draft. M. P. J., P. O. D., M. B., and N. F.: writing – review and editing.

## Conflicts of interest

The authors declare no competing interests.

## Supplementary Material
